# Novel PARP1/2 inhibitor mefuparib hydrochloride elicits potent *in vitro* and *in vivo* anticancer activity, characteristic of high tissue distribution

**DOI:** 10.18632/oncotarget.13749

**Published:** 2016-12-01

**Authors:** Jin-Xue He, Meng Wang, Xia-Juan Huan, Chuan-Huizi Chen, Shan-Shan Song, Ying-Qing Wang, Xue-Mei Liao, Cun Tan, Qian He, Lin-Jiang Tong, Yu-Ting Wang, Xiao-Hua Li, Yi Su, Yan-Yan Shen, Yi-Ming Sun, Xin-Ying Yang, Yi Chen, Zhi-Wei Gao, Xiao-Yan Chen, Bing Xiong, Xiu-Lian Lu, Jian Ding, Chun-Hao Yang, Ze-Hong Miao

**Affiliations:** ^1^ Division of Anti-Tumor Pharmacology and State Key Laboratory of Drug Research, Shanghai Institute of Materia Medica, Chinese Academy of Sciences, Shanghai 201203, China, University of Chinese Academy of Sciences, Beijing 100049, China; ^2^ Department of Medicinal Chemistry, State Key Laboratory of Drug Research, Shanghai Institute of Materia Medica, Chinese Academy of Sciences, Shanghai 201203, China, University of Chinese Academy of Sciences, Beijing 100049, China; ^3^ Shanghai Institute of Materia Medica, Chinese Academy of Sciences, Shanghai 201203, China; ^4^ Cisen Pharmaceutical Co., LTD, Jining 272073, Shandong, China

**Keywords:** MPH, PARP inhibitor, homologous recombination, antitumor activity, synthetic lethality

## Abstract

The approval of poly(ADP-ribose) polymerase (PARP) inhibitor AZD2281 in 2014 marked the successful establishment of the therapeutic strategy targeting homologous recombination repair defects of cancers in the clinic. However, AZD2281 has poor water solubility, low tissue distribution and relatively weak *in vivo* anticancer activity, which appears to become limiting factors for its clinical use. In this study, we found that mefuparib hydrochloride (MPH) was a potent PARP inhibitor, possessing prominent *in vitro* and *in vivo* anticancer activity. Notably, MPH displayed high water solubility (> 35 mg/ml) and potent PARP1/2 inhibition in a substrate-competitive manner. It reduced poly(ADP-ribose) (PAR) formation, enhanced γH2AX levels, induced G2/M arrest and subsequent apoptosis in homologous recombination repair (HR)-deficient cells. Proof-of-concept studies confirmed the MPH-caused synthetic lethality. MPH showed potent *in vitro* and *in vivo* proliferation and growth inhibition against HR-deficient cancer cells and synergistic sensitization of HR-proficient xenografts to the anticancer drug temozolomide. A good relationship between the anticancer activity and the PARP inhibition of MPH suggested that PAR formation and γH2AX accumulation could serve as its pharmacodynamic biomarkers. Its high bioavailability (40%~100%) and high tissue distribution in both monkeys and rats were its most important pharmacokinetic features. Its average concentrations were 33-fold higher in the tissues than in the plasma in rats. Our work supports the further clinical development of MPH as a novel PARP1/2 inhibitor for cancer therapy.

## INTRODUCTION

Genomic instability is an important characteristic of cancers [1, 2]. Defects in DNA damage response (DDR) including DNA repair are one of primary reasons for such instability [2, 3]. It has been shown that germline defects in 58 of 450 DDR genes are linked to inherited cancer predisposition or to cancer-associated syndromes [3]. It has been estimated that approximately half of high-grade serious ovarian cancer harbors homologous recombination repair (HR) defects that also occur in other cancers including breast, prostate and pancreatic cancer [2]. Therefore, targeting DNA-repair defects has been extensively investigated as an approach for cancer therapy [3, 4]. Poly(ADP-ribose) polymerases (PARPs) are a family of enzymes that catalyze the polymerization of ADP-ribose units from nicotinamide adenine dinucleotide (NAD^+^) on target proteins. Among this family, PARP1 and PARP2 are essential to base excision repair (BER) [5]. Inhibition of PARP has been undoubtedly the focus of this strategy in recent years [6]. Approval of the PARP1/2 inhibitor AZD2281 (also known as olaparib) for the treatment of BRCA-deficient ovarian cancer in 2014 marked the successful establishment of such a therapeutic strategy for cancer in the clinic [3].

However, although the current PARP inhibitors, including the approved one (AZD2281; also known as olaparib) and those at late-stage clinical trials, *i.e*., rucaparib (AG-014699), veliparib (ABT-888), niraparib (MK4827) and talazoparib (BMN673), can elicit potent PARP inhibition, particularly at molecular levels, all of them have very low water solubility (< 1 mg/ml) [7]. This feature might be unfavorable for making their formulations and for their absorption and distribution in the human body. As an example, the solubility of AZD2281 is only 0.1 mg/ml in aqueous media that is independent of pH [8]. Its licensed capsule formulation (50 mg) contains a relatively high proportion of surfactants, needs to be stored strictly at 15~30°C and even at this condition has a relatively short shelf-life of 1.5 years. Moreover, patients need to take 16 capsules each day (400 mg bid). AZD2281 has an average V_d_ of 40.3 L in humans [9], 1.18 L/kg in rats and 1.3 L/kg in dogs [10], indicating that tissues, especially its potentially-targeted tissues, has comparatively low drug exposure and concentrations. Moreover, AZD2281 is hard to go through the blood-brain barrier [9] and is a substrate of the drug transporter P-glycoprotein (P-gp) [11], further limiting its uses in some tumors, such as malignant glioma (a high proportion of which harbors HR defects due to PTEN mutations) and P-gp-overexpressed tumors. These characteristics of AZD2281 are not conducive to its clinical uses and are likely to restrict its therapeutic effectiveness.

**Figure 1:**
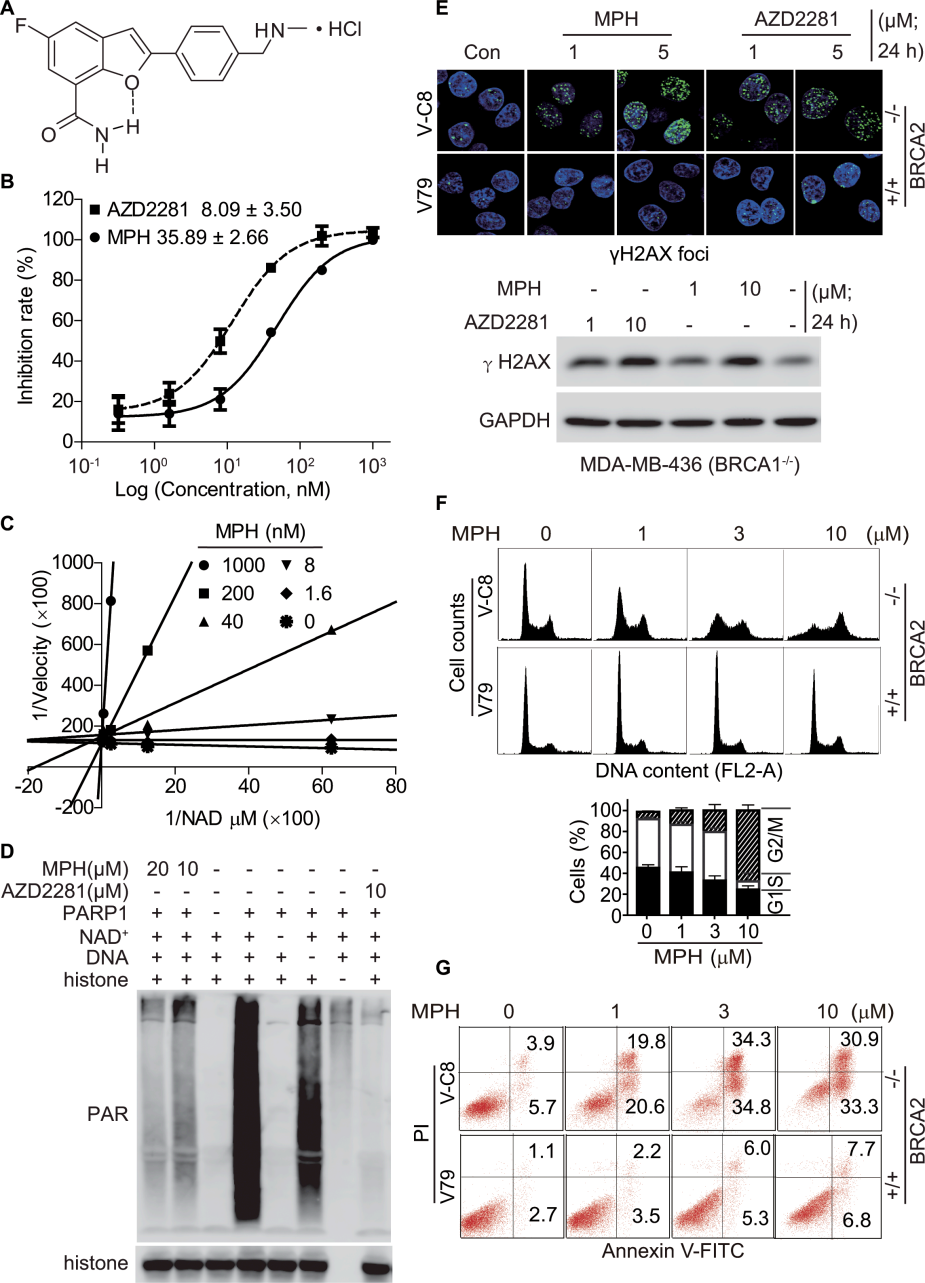
MPH inhibits PARP1 (**A**) chemical structure of MPH. (**B**) the concentration-effect relationships of PARP1 inhibition by MPH and the positive control AZD2281 assayed by ELISA. Insets: IC_50_ values. (**C**) the Lineweaver-Burk plots based on the reaction velocity at different concentrations of NAD^+^ and MPH measured by ELISA. (**D**) MPH or AZD2281 inhibited PARP1-catalyzed PAR formation in a cell-free system determined by Western blotting. (**E**) the changes in the formation of γH2AX foci in V-C8 and V79 cells (upper panel; confocal microscopy) and in the protein levels of γH2AX in MDA-MB-436 cells (lower panel; Western blotting) induced by MPH or AZD2281. (**F**) and (**G**), MPH selectively induced cell cycle arrest (F) and apoptosis (G) in HR-deficient cells. BRCA2-deficient (V-C8) and proficient (V79) cells were respectively exposed to MPH for 24 h (F) or 48 h (G) and then collected for PI-stained (F) or Annexin V-FITC-PI-stained (G) flow cytometry. All data were expressed as mean ± SD or representative images from 3 independent experiments.

Therefore, improving pharmaceutical properties such as water solubility of candidate drugs with sufficiently potent capability to inhibit PARP will be an important strategy for the development of new PARP inhibitors. Based on this consideration, huge efforts were made [12, 13] and finally, mefuparib hydrochloride (MPH; Figure 1A), a new potent, PARP 1/2 inhibitor with high water-solubility was obtained. Its preclinical study has been completed and its clinical trials will be conducted soon.

Here we present the major preclinical pharmacological results of MPH with special attention to its pharmacokinetics (PK)-pharmacodynamics (PD) relationships. MPH has excellent water solubility (> 35 mg/ml, 350-fold higher than that of AZD2281) and can be made into common tablet formulations. We show its PARP1/2 inhibition, selectivity, anticancer activity and PD biomarkers in both *in vitro* and *in vivo* models. We also report its PK characteristics including metabolic species differences, major PK parameters and tissue distribution, favorably supporting its potential therapeutic uses.

## RESULTS

### MPH is a potent inhibitor of PARP1 and PARP2

MPH has a novel chemical structure designed by using benzofuran as a core structure *via* a privileged structure strategy and adopting an intramolecular hydrogen bond (pseudo bicyclic ring) instead of a fused amide bond. MPH has excellent water solubility (> 35 mg/ml) and stability (no detectable changes for more than 2 years at room temperature). MPH showed potent inhibition against PARP1 [IC_50_: 35.89 nM (Figure 1B; ELISA assays) or 3.2 nM (Supplementary Table S1; biotinylated NAD^+^-based assays)] and PARP2 [IC_50_: 1.9 nM (Supplementary Table S1)]. It revealed high selectivity of PARP1/2, more than 406 fold over other major nuclear PARPs including PARP3, TNKS1, TNKS2 and PARP6 (Supplementary Table S1). Though MPH inhibited PARP1/2 about 2~4-fold less potently than the approved inhibitor AZD2281, it displayed much higher selectivity of PARP1/2 over the other examined PARP family members (Figure 1B; and Supplementary Table S1).

Mechanistic studies indicated that MPH inhibited the catalytic activity of PARP1 in a substrate (NAD^+^)-competitive manner (Figure 1C) and thus reduced the formation of the resulting PAR (Figure 1D). Chinese hamster V-C8 cells have an impaired capacity of the HR pathway due to a deficiency in BRCA2 [21–23]. Relative to wild-type V79 cells, V-C8 cells are extremely sensitive to PARP inhibitor [22]. Furthermore, the treatments with MPH, just as with AZD2281, caused the accumulation of DSB marked by the increased levels of γH2AX in the BRCA-deficient V-C8 (BRCA2^−/−^) and MDA-MB-436 (BRCA1^−/−^) cells in a concentration-dependent manner, but not in the BRCA-proficient V79 cells (Figure 1E). When exposed to gradient concentrations of MPH, consequently, V-C8 cells but not V79 cells came into typical G2/M arrest (Figure 1F) and subsequent apoptosis (Figure 1G).

All these data collectively indicate that MPH is a potent inhibitor of PARP1/2 with excellent structural novelty and water solubility.

### MPH elicits selective killing in HR-deficient cells both *in vitro* and *in vivo*: a proof of concept

**Figure 2:**
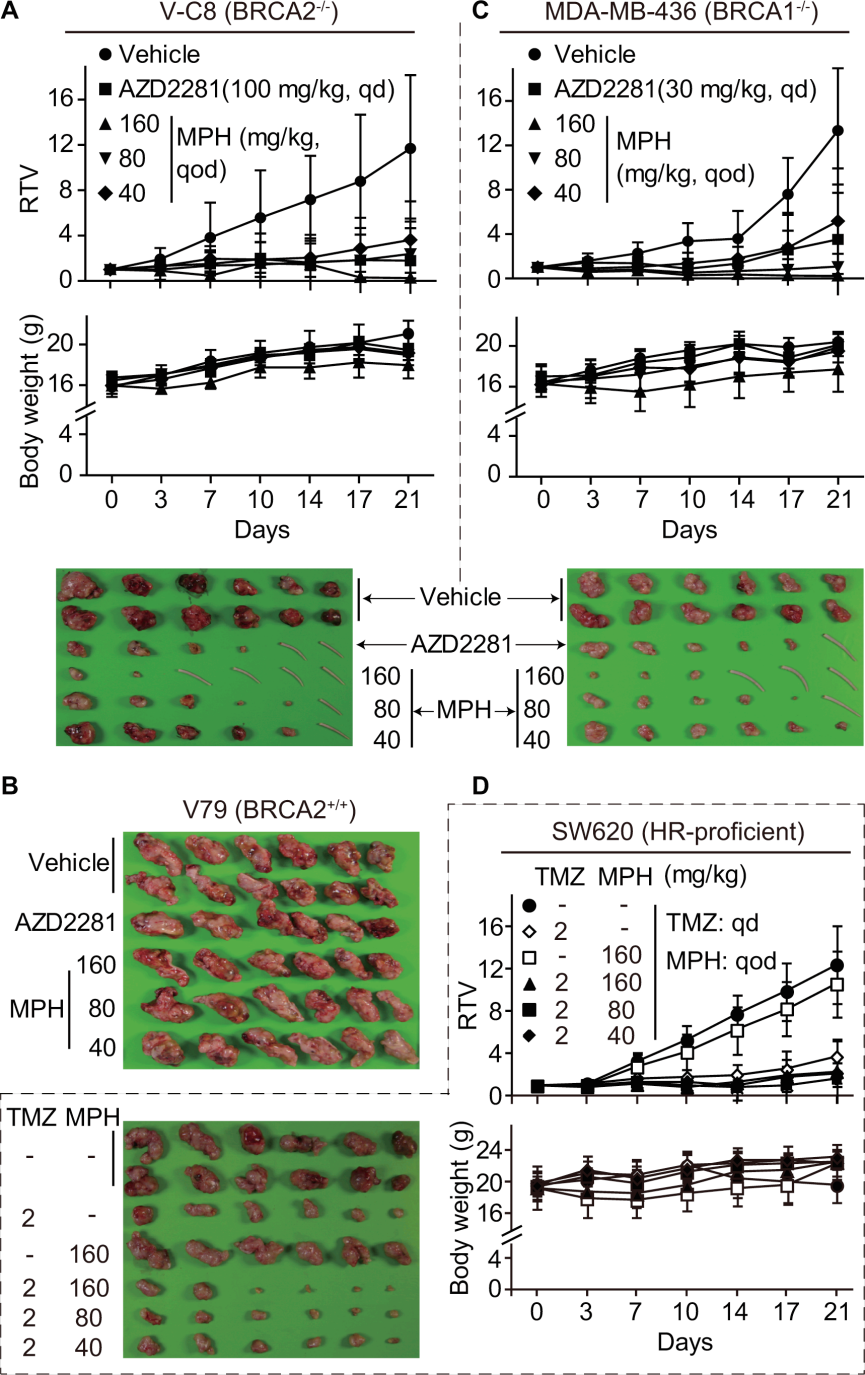
MPH selectively suppresses the growth of HR-deficient xenografts and sensitizes HR-proficient xenografts to the DNA damaging agent TMZ in nude mice The mice bearing subcutaneous xenografts were orally given MPH once every other day and/or TMZ once each day on Monday to Friday every week, either alone or in combination. Relative tumor volume (RTV) and body weight were separately plotted over the treatment time. The xenografts or the mice tails if the xenografts disappeared in the corresponding mice were separately taken for photographing at the end of treatments. (**A**–**C)** the effect of MPH alone on BRCA2-deficient V-C8 (A), BRCA2-proficient V79 (B) and BRCA1-deficient MDA-MB-436 (C) xenografts. (**D**) the effect of MPH and TMZ, alone or in combination, on HR-proficient SW620 xenografts.

PARP1/2 inhibition causes synthetic lethality in HR-deficient cells [22]. To further conduct proof-of-concept studies with MPH, we used a well-characterized isogenic system consisting of Chinese hamster lung fibroblast cells: V-C8 (BRCA2^−/−^), V79 (wild-type) and V-C8+H13 (BRCA2 genetically complemented with the 13^th^ chromosome) cells [23]. *In vitro* assays showed that MPH elicited cell killing in V-C8 46.85- and 97.56-fold more potently than in V79 and V-C8+H13 cells, respectively. By contrast, AZD2281 caused 25.64- and 22.31-fold more potent cell killing in the BRCA2^−/−^ cells than in V79 and V-C8+H13 cells, respectively, indicating that MPH has higher selectivity than AZD2281 in this case (Table 1). In nude mice subcutaneous xenograft models, consistently, MPH displayed dose- and time-dependent killing on V-C8 xenografts accompanied by complete disappearance of some xenografts, especially in the high-dose group. The positive control AZD2281 revealed similar killing, and its effect at 100 mg/kg each day was between those of MPH at 80 mg/kg and 180 mg/kg every other day. At all the tested doses, MPH or AZD2281 did not cause death or significant body-weight loss of the animals during the experiment (Figure 2A). In sharp contrast, the similar treatments with MPH or AZD2281 did not inhibit the growth of V79 xenografts (Figure 2B). The data prove the concept that MPH can cause the synthetic lethality selectively in HR-deficient cells.

**Table 1:** Proof of concept and selective inhibition of MPH against the proliferation of cells harboring deficient BRCA1, BRCA2, PTEN or EWS-FLI1

Cell lines	Types	Mutations	IC_50_ (mean ± SD) (μM)		IC_50(AZD2281)_/IC_50(MPH)_
			MPH	AZD2281*	
V-C8	Chinese hamster	BRCA2^−/−^	0.54 ± 0.18	0.61 ± 0.04	1.13
V79	lung fibroblasts	BRCA2^+/+^	25.30 ± 1.68	15.64 ± 1.15	0.62
V-C8+H13		BRCA2^+/+^	52.68 ± 3.06	13.61 ± 1.85	0.26
MDA-MB-436	breast cancer	BRCA1^−/−^	0.12 ± 0.01	0.02 ± 0.01	0.17
HCC1937	breast cancer	BRCA1^−/−^	3.61 ± 0.47	7.28 ± 1.91	2.02
UWB1.289	ovarian cancer	BRCA1^−/−^	1.73 ± 0.25	2.98 ± 1.65	1.72
Capan-1	pancreatic cancer	BRCA2^−/−^	2.35 ± 0.16	1.73 ± 0.32	0.74
DoTc2-4510	cervical cancer	BRCA2^−/−^	1.91 ± 0.38	0.89 ± 0.28	0.47
HCT-15	colon cancer	BRCA2^−/−^	2.64 ± 0.59	25.70 ± 4.70	9.73
U-87 MG	glioblastoma	PTEN^−/−^	2.89 ± 0.62	13.13 ± 2.34	4.54
U251	glioblastoma	PTEN^−/−^	3.64 ± 0.80	2.50 ± 0.64	0.69
PC-3	prostate cancer	PTEN^−/−^	2.52 ± 0.30	4.70 ± 0.69	1.87
SK-ES-1	Ewing sarcoma	EWS-FLI1	1.82 ± 0.27	1.70 ± 0.13	0.93
RD-ES	Ewing sarcoma	EWS-FLI1	0.57 ± 0.14	0.67 ± 0.14	1.18

* The only approved PARP1/2 inhibitor was used as the positive control.

To clarify whether the anticancer activity of MPH is dependent on its PARP1 inhibition, we constructed a PARP1-deficient cell line using the transcription activator-like effector nuclease technique (TALEN) technique in Ewing sarcoma RD-ES cells (Supplementary Figure S1A). As expected, the knockout of PARP1 impaired the cellular sensitivity to MPH. The IC_50_ value of MPH increased 5-7 fold in the PARP1-deficient RD-ES cells when compared with that in the corresponding wild-type cells and the similar changes were seen with AZD2281 (Supplementary Figure S1B). Our data indicated that MPH inhibits the growth of HR-deficient cancer dependent on its PARP inhibition activity.

### MPH inhibits growth and proliferation of HR-deficient cancer cells and sensitizes HR-proficient cancer cells to anticancer drugs

We then examined the ability of MPH to suppress growth and proliferation of 11 cancer cell lines harboring well-characterized different deficiencies in the HR pathways, including BRCA1^−/−^, BRCA2^−/−^, PTEN^−/−^ and EWS-FLI1 [24] (Table 1). MPH exerted potent *in vitro* proliferation-inhibitory effects on these cancer cells derived from different human tissues with an average IC_50_ of 2.16 μM (0.12 μM ~ 3.64 μM), which is 2.58-fold more potent than AZD2281 (Table 1). Similarly, in the *in vivo* MDA-MB-436 (BRCA1^−/−^) xenograft model, MPH significantly inhibited the growth of the xenografts in dose- and time-dependent manners with complete disappearance of 1/2 xenografts in the high-dose group. In the same model, the effect of the positive control AZD2281 at 30 mg/kg each day was between those of MPH at 40 mg/kg and 80 mg/kg every other day (Figure 2C). The results indicate that MPH possesses the potential to kill human HR-deficient cancers. We further evaluated the potentiated efficacy of MPH on DNA damaging agent temozolomide (TMZ) *in vivo*. MPH alone even at 160 mg/kg every other day did almost not affect the growth of HR-proficient SW620 xenografts in nude mice. When combined, it potentiated the growth-inhibitory effects of TMZ (2 mg/kg) (Figure 2D). Specifically, the combination groups of 160 mg/kg and 80 mg/kg revealed more than 1 of combination ratios (2.8 and 2.0, respectively) (Supplementary Table S2), indicating that MPH is synergistic with TMZ in their *in vivo* anticancer activities.

**Figure 3:**
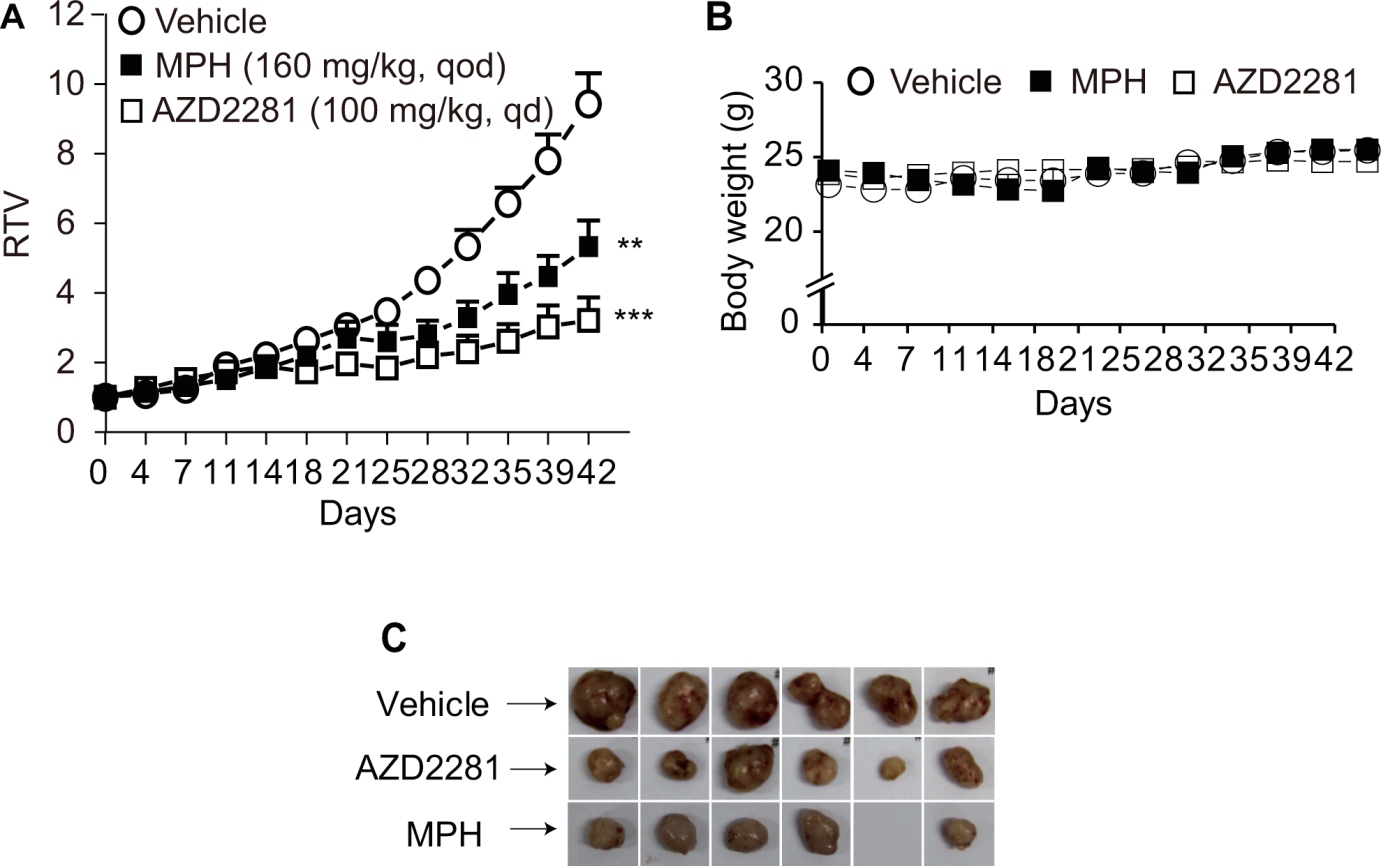
The *in vivo* anti-tumor efficacy of MPH in breast cancer patient-derived xenograft (PDX) model The nude mice bearing subcutaneous xenografts derived from breast cancer tissue of a patient carrying BRCA1 gene mutations were orally given MPH once every other day for consecutive 6 weeks. AZD2281 was used as the positive control. Relative tumor volume (RTV) (**A**) and body weight (**B**) were separately plotted over the treatment time. The images represented the xenografts at the end of treatments (**C**). The blank represented a disappeared tumor xenograft due to the treatment with MPH. Data were analyzed by Student *t* test. **P < 0.01; ***P < 0.001.

To further evaluate the *in vivo* anti-tumor efficacy of MPH, a subcutaneous BR-05-0028 breast patient-derived xenograft (PDX) model was used to test the treatment regimen of MPH as a single agent (BR-05-0028 with BRCA1 mutated at exon10:c.C1630T:p.Q544X; established and kept by WuXi AppTec, Shanghai, China). When given at 160 mg/kg once every other day, MPH inhibited the growth of the PDX without obvious loss of body weight (Figure 3A–3C).

Together, those data reveal the anticancer potential of MPH alone or in combination.

### MPH suppresses PAR formation in HR-deficient Capan-1 cells and MDA-MB- 436 xenografts: Pharmacodynamic analyses

**Figure 4:**
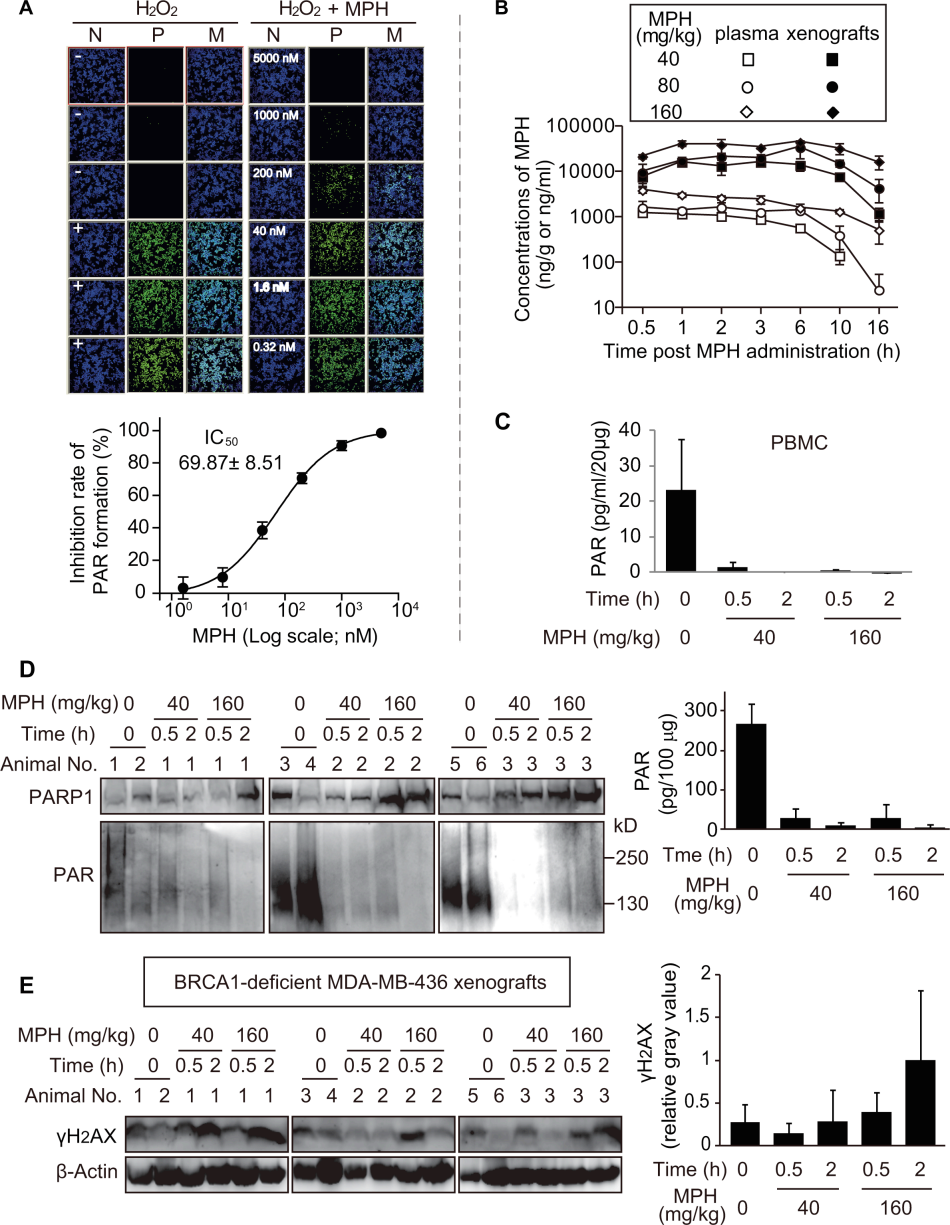
Pharmacodynamic biomarker analyses reflecting the *in vitro* and *in vivo* activities of MPH (**A**) MPH prevented the H_2_O_2_-triggered formation of PAR in BRCA2-deficient Capan-1 cells. Upper: representative images of PAR formation. Nuclei stained with DAPI indicated the cell density; the FITC images revealed the levels of cellular PAR. N, nuclei; P, PAR; M, merged. Lower: The concentration-response curve quantitatively showed the inhibition of PAR formation by MPH. The data were expressed as mean ± SD, obtained from the upper images and from three independent experiments. (**B**) the time-concentration curve of MPH in plasma and xenografts following a single-dose oral administration to the nude micebearing BRCA1-deficient MDA-MB-436 subcutaneous xenografts (The same models were also used in (**C**–**E**). C, the levels of PAR formation in peripheral-blood mononuclear cells (PBMC) assayed by ELISA. Data were presented as mean ± SEM showing a representative data from two independent experiments, and three animals in each group. D and E, the levels of PAR (D) and γH2AX (E) in MDA-MB-436 cells of the xenografts determined by Western blotting. Left, representative images; right, semi-quantitative results (mean ± SD) from the corresponding images. Six animals in vehicle group, three animals in each MPH treated group.

In the nucleus, the majority of PAR formation is catalyzed by PARP1/2 [25]. We have shown above that MPH is a PARP1/2 inhibitor, displaying its *in vitro* and *in vivo* anticancer activity. To further confirm the PARP1/2 target engagement following the exposure of MPH, we conducted pharmacodynamic analyses at cellular and xenograft levels. The exposure of BRCA2-deficient pancreatic cancer Capan-1 cells to the DNA damaging agent hydrogen peroxide (H_2_O_2_) induced large amounts of PAR formation in the cells (the upper left panel, Figure 4A). Pretreatments with MPH inhibited the H_2_O_2_-induced PAR formation in a concentration-dependent manner with an average IC_50_ of 69.87 nM, and when at concentrations > 1 μM, MPH could completely prevent the formation of PAR (the upper right panel and the lower panel, Figure 4A). The data indicate that MPH could inhibit the catalytic activity of its molecular targets PARP1/2 in the cellular system. This conclusion is further strengthened by its direct inhibition on PAR formation in the cell-free system (Figure 1D) and its induction of DSB accumulation in HR-deficient V-C8 and MDA-MB-436 cells (Figure 1E).

To do the *in vivo* pharmacodynamic analyses, we determined the concentrations of MPH in the plasma and MDA-MB-436 xenografts in nude mice at different times post oral administration of MPH at 40, 80 or 160 mg/kg (Figure 4B). The doses were the same as those used to test its *in vivo* anticancer activity (Figure 2). Its concentrations were shown to have good dose- and time-dependency. The concentrations in the plasma rapidly reached the peak following the MPH administration (T_max_: 0.5~2 h) but later in the xenografts (T_max_: 3~6 h). However, the exposure of MPH in the xenografts was above 20-fold higher than that in the plasma in each dose group (Supplementary Table S3). Consistently, at the same time-point and the same dose, the concentrations in the xenografts were higher than those in the plasma (Figure 4B). At 0.5 h, the concentrations of MPH in the groups of 40, 80 and 160 mg/kg were 1274, 1626 and 3939 ng/ml in the plasma and 7740, 9667 and 21786 ng/g in the xenografts, respectively. They were approximately equivalent to 3.78, 4.83 and 11.70 μM in the plasma and 22.98, 28.70 and 64.69 μM in the xenografts. In contrast, at 2 h, its concentrations were 1058 (3.14), 1655 (4.91) and 2686 (7.98) ng/ml (μM) in the plasma and 13242 (39.32), 21457 (63.71) and 39139 (116.21) ng/g (μM) in the xenografts, respectively (Figure 4B). Therefore, at these two time points, all the determined concentrations of MPH were higher than that at which MPH could inhibit PARP1/2.

We thus evaluated PARP inhibition at 0.5 and 2 h following MPH administration in the low (40 mg/kg) and high (160 mg/kg) -dose groups by functional assays for PAR formation in peripheral-blood mononuclear cells (PBMC) and MDA-MB-436 xenograft tumor cells in nude mice. In both PBMC and xenograft tumor cells, MPH resulted in rapid and dramatic inhibition of PARP, as shown by more than 90% reduction at 0.5 h and almost complete disappearance of PAR formation at 2 h (Figure 4C and 4D). Consistently, in the BRCA1-deficient MDA-MB-436 xenograft tumor cells, the levels of γH2AX, a DSB marker, increased in time- and dose-dependent manners (Figure 4E). The results indicate that at the *in vivo* experimental doses, MPH could rapidly reach the effective therapeutic concentrations and lead to significant PARP inhibition, which is quickly coupled with downstream induction of DSB in xenografts.

### Pharmacokinetic characteristics of MPH in animals

**Figure 5:**
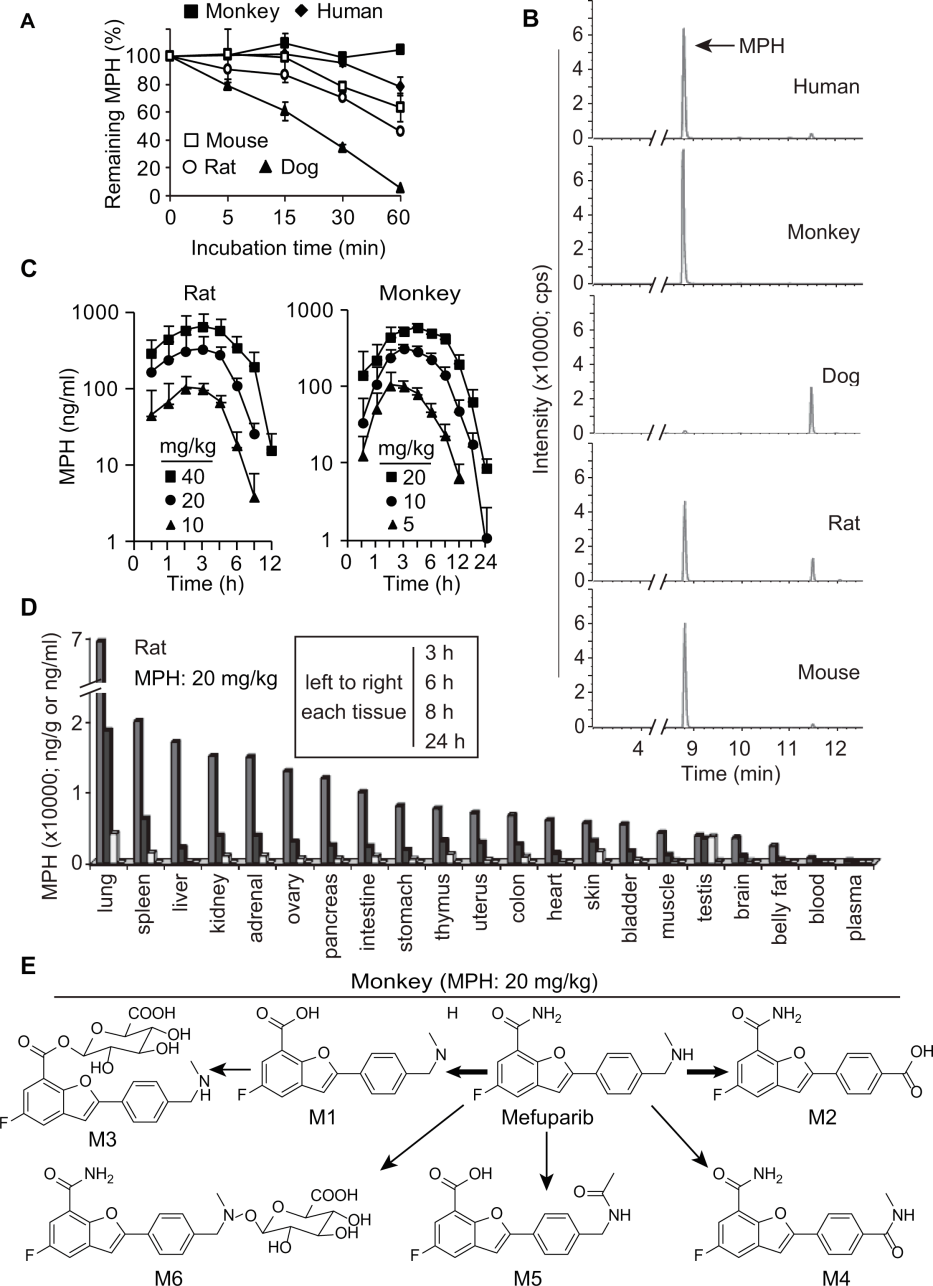
Pharmacokinetics of MPH (**A**) and (**B**) the *in vitro* metabolic stability (A) and metabolite profile (B) of MPH in liver microsomes from different species of animals. Data were presented as mean ± SEM showing a representative data from two independent experiments. (**C**) the time-concentration curves of MPH in plasma following a single-dose oral administration to rats (left) and monkeys (right). (**D**) the concentrations of MPH in different tissues following 20 mg/kg oral administration to rats. (**E)** the proposed metabolic pathway of MPH in monkeys. Three animals in each group.

To choose proper animals to investigate the *in vivo* pharmacokinetic characteristics of MPH, we compared its metabolic stability and its metabolite profile in liver microsomes from different species of animals, including mouse, rat, dog and monkey with those in human liver microsomes. The data showed that MPH was the stablest in the monkey liver microsomes with a remaining amount of about 90% and the most instable in the dog liver microsomes with nearly no remaining amount after 60-min incubation. In human, rat and mouse liver microsomes, MPH following 60-min incubation remained 78.5%, 46.3% and 63.6%, respectively (Figure 5A). This phenomenon was further reflected in its metabolite profile: after the same-time incubation in liver microsomes, its metabolites increased by the order of monkey, human, mouse, rat and dog although their metabolite spectrums were similar overall (Figure 5B). Primarily based on these data, we selected rat and monkey to the subsequent pharmacokinetic studies.

Following its single-dose oral administration of different doses in both rat and monkey (Figure 5C; Supplementary Table S4), MPH was rapidly absorbed with average plasma T_max_ of 2~4 h. The plasma concentrations and exposure increased over the increasing doses. Importantly, the plasma C_max_ in both animals at the doses of 20 mg/kg or above was higher than 1 μM, a concentration at which MPH could almost completely inhibit the catalytic activity of PARP1/2 (Figure 4A). MPH had high bioavailability (above 40%) (Supplementary Table S4, S5) and seemed to increase over the increasing doses, possibly because its metabolism was saturated at higher doses. MPH displayed a longer average t_1/2_ in monkeys (2.5 h) than in rats (1.2 h) and its plasma concentrations could not be detected after 24 h in monkeys but within 12 h in rats, indicating its slower elimination in the former (Figure 5A and 5B).

In rats after being given 20 mg/kg orally, MPH was extensively distributed to all the 18 detected tissues, in which its concentrations were average-33-fold higher than that in the plasma at the 3 h -time point post its administration (Figure 5D). Among these tissues compared with the plasma at 3 h, lung got the highest MPH concentration (188-fold) and the fat tissue had the lowest (7-fold); the concentration of MPH in the brain also reached a high level of 10-fold, indicating that MPH easily enters into the brain tissue through the blood-brain barrier. Notably, the tissues corresponding to those in human, from which cancers harboring HR-deficiency have been reported, including ovary, pancreas, colon and uterus, also had high MPH distribution, higher than that in the plasma by 35, 33, 18, and 19 times, respectively (Figure 5D). MPH had its T_max_ of 3 h post its administration, and then reduced over the time, with average concentrations of 28%, 8% and lower than 1% of its C_max_ at 6-h, 8-h and 24-h time points, respectively.

Following its oral administration at 10 mg/kg in monkeys, 6 metabolites of MPH could be detected (Figure 5E). M1 and M2, which were respectively generated *via* amide hydrolysis and oxidative deamination of MPH, were its 2 major direct metabolites, accounting for 52.18% and 1.86% of the total amount of all the metabolites and MPH itself in the plasma at the 3-h time point. M3 (a secondary metabolite of M1 *via* glucuronate conjugation) to M6 accounted for 4.18%, 0.24%, 0.72% and 1.63% at the same time point, respectively. At the 12-h time-point post the MPH administration, only MPH itself, M1 and M2 could be detectable in the plasma, with average remaining amounts of 6.79%, 3.13% and 23.54% of their corresponding plasma amount at 3 h, respectively. MPH that was not metabolized and its metabolites were basically excreted *via* feces and urine.

The above data show that MPH possesses good pharmacokinetic characteristics in animals, and especially its high distribution in the potentially therapeutically- targeted tissues greatly favors the treatment of HR-deficient cancers with MPH.

## DISCUSSION

We here present MPH as a novel PARP1/2 inhibitor with good pharmacokinetic characteristics. MPH has a novel chemical structure consisting of a privileged structure benzofuran carrying an intramolecular hydrogen bond to keep a pseudo bicyclic pharmacophore. MPH possesses high stability and appropriate lipophilicity, and the salification with hydrochloride confers its high water solubility. These features offer its high bioavailability and facilitate to make its formulations for uses in humans, which confer prominent advantages upon MPH especially in considering the related problems of the majority of PARP inhibitors currently approved or under late-stage clinical trials arising from their poor water solubility.

MPH displays potent PARP1/2 inhibition, which can be effectively translated into its biological effects including reducing *in vitro* and *in vivo* PAR formation, and inducing DSB (marked by γH2AX) accumulation and subsequently causing cell cycle arrest and apoptosis in HR-deficient (*e.g*., V-C8 and MDA-MB-436) rather than HR-proficient (*e.g*., V79) cells. Although at molecular levels, MPH inhibits PARP1/2 2-4-fold less potently than the approved PARP inhibitor AZD2281, it causes 2.58-fold more potent *in vitro* proliferative inhibition than the latter in HR-deficient cancer cells and approximately equipotent *in vivo* growth inhibition in HR-deficient xenografts. This difference possibly arises from better solubility and higher permeability of MPH. The difference is further supported by the fact that oral administration of MPH yields higher concentrations and greater exposure (more than 20-fold) in the xenografts than in the plasma in MDA-MB-436 xenograft models. At this condition, PARP1/2 in PBMC and tumor cells in the xenografts can be inhibited nearly totally and DSB accumulation progressively increases in tumor cells in the xenografts, which forms the basis of the *in vivo* anticancer activity of MPH.

MPH shows better selectivity at either molecular or cellular levels than AZD2281. This selectivity can be further turned into single-agent growth inhibition of MPH in the HR-deficient xenografts (*e.g*., V-C8 and MDA-MB-436) but not in the HR-proficient xenografts (*e.g*., V79 and SW620). Additionally, MPH in combination with TMZ elicits synergistic growth inhibition on HR-proficient SW620 xenografts in nude mice. In fact, the synergism of MPH with TMZ is similar to that of other PARP inhibitors under clinical trials [11]. All the results suggest that MPH has the potential to exert more selective anticancer effects than AZD2281 in the clinic.

MPH also shows prominent advantages in its pharmacokinetics. MPH can be absorbed nearly completely with high bioavailability (40%~100%). Its distribution is very wide and in rats, its average concentrations in the tissues can be 33-fold higher than those in the plasma. Especially importantly, the potential tissues therapeutically targeted by MPH including ovary, pancreas, colon and uterus also have much greater concentrations than the plasma. Relatively slow metabolism in monkeys that have the most similar metabolic characteristics to humans also favors cancer therapy with MPH. Therefore, these pharmacokinetic features lay an excellent basis for the therapeutic uses of MPH in humans.

Collectively, MPH is a new PARP1/2 inhibitor with high water solubility and structurally different from all the PARP inhibitors approved and in clinical trials. Its potent PARP1/2 inhibition can be effectively translated into its selective killing on HR-deficient cancers and synergistic sensitization of HR-proficient cancers to anticancer drugs. Its excellent solubility facilitates to make its formulations and favors its oral absorption. Its high distribution in tissues and especially in those potentially targeted tissues further supports its potential anticancer uses. Together, our results indicate that MPH is promising in its future clinical development.

## MATERIALS AND METHODS

### Synthesis and purification of MPH

Mefuparib employed privileged structure benzofuran as the scaffold and utilized an intramolecular hydrogen bond to mimic the fused amide bond as the bioactive pharmacophore [14]. The free base of mefuparib (5-fluoro-2-(4-((methylamino) methyl)phenyl)benzofuran-7-carboxamide; Figure 1A) was synthesized from methyl 5-fluoro-3-iodosalicylate and 1-(4-ethynyl)-N-methylmethanamine in two steps. Methyl 5-fluoro-2-(4-((methylamino)methyl)phenyl)benzofuran-7-carboxylate was prepared by a cascade reaction in the presence of Pd(PPh_3_)_2_Cl_2_/CuI and then ammonolyzed with ammonia methanol solution to give the target compound [15]. The free base was converted to monohydrochloride in ethanol with concentrated hydrochloric acid and then recrystallized to give the pure product MPH (> 98.4%, HPLC). MPH has high water solubility (> 35 mg/mL) and appropriate lipophilicity (ClogP 2.99). Details of the design and structure-activity relationships for MPH and its analogs will be reported elsewhere soon.

### Drugs, reagents and antibodies

AZD2281 was purchased from LC Laboratories (Woburn, MA). Temozolomide (TMZ) was from Jiang Su Tasly Diyi Pharmaceutical Co., Ltd (Huaian, Jiangsu, China). The drugs were dissolved in dimethyl sulfoxide (DMSO) and stored at −20°C. Z-VAD.fmk, JC-1, RNase and propidium iodide (PI) were from Beyotime Institute of Biotechnology (Haimen, Zhejiang, China). All other chemical reagents were from Sigma (St. Louis, MO).

Antibodies against β-actin and caspase-3 were from Cell Signaling Technology (Beverly, MA), and antibodies against PAR, γH2AX and histone H3 came from Santa Cruz Biotechnology (Santa Cruz, CA).

### Cell culture

Chinese hamster cell lines V-C8, V79 and V-C8+H13 were gifts from Prof M Zdzienicka (Leiden University, Amsterdam, The Netherlands). Human glioblastoma cell lines U-87 MG and U251 were purchased from the Shanghai Institutes for Biological Sciences of the Chinese Academy of Sciences. All other cell lines were from the American Type Culture Collection. The cells were periodically authenticated by morphologic inspection and tested for Mycoplasma contamination. The cells were cultured according to the suppliers’ instructions.

### PARP inhibition assays in cell-free systems

The inhibition of the tested compounds on PARP1 enzymatic activity in a cell-free system was determined by ELISA as reported previously [13]. A similar assay in which NAD^+^ was used at gradient concentrations was done to analyze whether MPH inhibited PARP1 in a substrate (NAD^+^)-competitive manner. And a similar but amplified 100-ml reaction system containing 100 μg/ml PARP1, 800 μM NAD^+^, 30 μg/ml DNA and 1 mg/ml histone was used to detect the inhibition of PAR formation by the standard Western blotting. The selective inhibition of the tested drugs on the PARP family members including PARP1, PARP2, PARP3, TNKS1, TNKS2 and PARP6 was measured by biotinylated NAD^+^-based luminescence assays by BPS Bioscience (San Diego, CA).

### Assays for γH2AX accumulation in cultured cells

γH2AX accumulation in the cells exposed to MPH or AZD2281 was assayed by immunofluorescence-based laser confocal microscopy or Western blotting as described previously [16].

### Flow cytometry

V-C8 and V79 cells following treatments with MPH for 24 h (for cell cycle arrest) or 48 h (for apoptosis) were analyzed by PI-staining-based (for cell cycle arrest) or AnnexinV-FITC and PI-staining-based (for apoptosis) flow cytometry as described previously [16, 17].

### Proliferative inhibition assays

Cells cultured in 96-well plates were treated with PARP inhibitors alone or in combination with the indicated anticancer drugs for 3 days (Chinese hamster cells and Ewing sarcoma cells) or 7 days (all the other cells). Then the cells were subjected to the Cell Counting Kit 8 (CCK8) assays (Chinese hamster cells, Ewing sarcoma cells, HCC1937 and MDA-MB-436 cells) or by Sulforhodamine B (SRB) assays (all the other cells) as described previously [18, 19]. The inhibition rate (%) was calculated as: [1-(A450_treated_/A450_control_)] × 100%. The averaged IC_50_ values (mean ± SD) were determined with the Logit method from three independent tests.

### *In vivo* anticancer activity experiments

V-C8, V79, MDA-MB-436 and SW620 xenografts were established by inoculating 5 × 10^6^ cells s.c. in nude mice, respectively. After 1~2 passages in nude mice, the well-grown xenografts were cut into 1.5-mm^3^ fragments that subsequently were transplanted s.c. into the right flank of nude mice. When xenografts reached a volume of 100–200 mm^3^ (230-240 mm^3^ for V79), the mice were randomized to control and treated groups and received the corresponding treatments as indicated. The doses and the administration time interval of MPH were determined according to its preliminary experimental results that showed more potent growth inhibition of MPH in the V-C8 xenograft models when the same total dosing amount of MPH was orally administrated once every other day rather than once each day. The size of xenografts and the body weight of mice were measured individually twice per week. The xenografts were measured for the maximal width (X) and length (Y) and the volume (V) was calculated as (X^2^Y)/2. Relative tumor volume (RTV) was calculated as V_t_ / V_0_, where V_0_ and V_t_ were the volume before and after treatments, respectively. The *in vivo* anticancer activity was judged by their T/C (%) values. T/C (%) was calculated as (T_RTV_ / C_RTV_)×100%, where T_RTV_ and C_RTV_ represented the RTV of the treatment and the vehicle group, respectively.

The anticancer activity of MPH against patient-derived xenografts (PDX) was determined by WuXi AppTec (Shanghai, China). The human breast PDX BR-05-0028, containing a BRCA1 gene mutation [BRCA1 (NM 007294): exon10 C1630T (Q544X)]. When the xenografts grew to approximately 150 mm^3^, the animals were randomized into various groups and treated as indicated.

The experiments abided by institutional ethical guidelines of the Animal Care and Use Committee in our institute.

### *In vitro* and *in vivo* pharmacodynamic studies

In the *in vitro* pharmacodynamic assay, *brca2*-deficient Capan-1 cells were grown on 96-well black microplates at the density of 10^4^ cells/well. The cells were pretreated with MPH and treated with H_2_O_2_ as indicated and then analyzed for the intracellular PAR formation at an IN Cell Analyzer 2000 (GE Healthcare) just as described previously [13].

The *in vivo* pharmacodynamic assay was conducted by using a *brca1*-deficient MDA-MB-436 xenografts model in nude mice as described above. After the single-dose oral administration of MPH, the blood and xenografts of mice were taken for determining the concentrations of MPH and the levels of PAR formation and/or γH2AX accumulation at the indicated time points. The liquid chromatography-mass spectrometry/mass spectrometry (LC-MS/MS) was used to measure the concentrations of MPH in the plasma and xenografts of mice. The levels of PAR formation in peripheral-blood mononuclear cells (PBMC) and tumor cells in the xenografts were evaluated by using the similar protocols as reported previously and all were normalized to the amount of PARP1 protein present [9]. The levels of γH2AX accumulation in tumor cells in the xenografts were determined by Western blotting and β-actin was used as the internal control.

### Pharmacokinetic studies

The metabolic stability and metabolite profiles of MPH were analyzed by using liver microsomes of different species (BD Gentest, Woburn, MA) as indicated. Assays were conducted in a 100-μl volume containing 100 mM potassium phosphate buffer (PBS, pH7.4), 0.5 mg/ml liver microsomal protein (the corresponding inactivated liver microsomal protein was used in the control group), 3.0 μM MPH and 1.0 mM NADPH. Following the incubation at 37°C for the indicated time (for the metabolic stability analysis) or 60 min (for the metabolite profile analysis), reactions were terminated by addition of 100 μl ice-cold acetonitrile. LC-MS/MS was used to measure the remaining MPH in the metabolic stability analysis while UPLC-UV/Q-TOF MS was used to identify the metabolite profile of MPH as described previously [20].

SD rats and cynomolgus monkeys were used to do the *in vivo* pharmacokinetic studies. Following MPH administration, blood and tissues were collected at the indicated time and analyzed for the MPH concentration by LC-MS/MS. The plasma MPH concentration-time data were analyzed with the noncompartmental method (Phoenix, version 1.3; Pharsight, Mountain View, CA) to derive pharmacokinetic parameters. The metabolites in the monkey plasma were analyzed by UPLC-UV/Q-TOF MS. The metabolite data were collected with Analyst^®^ TF V1.6 (AB Sciex Inc., Framingham, MA) and Masslynx V4.1 (Waters Corporation, Milford, MA) and analyzed with PeakView^®^ V1.2 and MetabolitePilot V1.5 (AB Sciex).

### Statistical analyses

All the data were presented as mean ± SD. The Student's *t-test* was used to determine the significant difference between the drug-treated group and the control group. *P <* 0.05 was considered to be significant statistically.

## SUPPLEMENTARY TABLES AND FIGURE


