# Supplementary material for: Novel PARP1/2 inhibitor mefuparib hydrochloride elicits potent in vitro and in vivo anticancer activity, characteristic of high tissue distribution
Source: Oncotarget. 2016 Dec 1;8(3):4156–68. doi: 10.18632/oncotarget.13749 (PMC5354820; doi:10.18632/oncotarget.13749)
Supplement: Supplementary file 1 [file oncotarget-08-4156-s001.pdf]

Novel PARP1/2 inhibitor mefuparib hydrochloride elicits potent *in vitro* and *in vivo* anticancer activity, characteristic of high tissue distribution

Supplementary Materials

Supplementary Table S1: The selective inhibition of MPH against PARPs analyzed by biotinylated NAD^+-based luminescence assays

PARPs	MPH		AZD2281		XAV939
	IC_{50} (nM)	Fold*	IC_{50} (nM)	Fold*	IC_{50} (nM)
PARP1	3.2	1.00	1.1	1.00	—
PARP2	1.9	0.59	0.9	0.82	—
PARP3	> 10000	> 3125.00	258	234.55	—
TNKS1	1600	500.00	—	—	14.5
TNKS2	1300	406.25	—	—	5.9
PARP6	> 10000	> 3125.00	1500	1363.64	—

NOTE: XAV939 is an inhibitor for TNKS1 and TNKS2.
*Fold= $IC_{50\,(PARP)}/IC_{50\,(PARP1)}$.

Supplementary Table S2: MPH potentiated the cytotoxicity of temozolomide (TMZ) in the nude mice SW620 xenograft model

MPH (d21)	FTV[a]		Combination		
	MPH	TMZ	Expected FTV[b]	Observed FTV[c]	Combination ratio[d]
160 mg/kg	0.88	0.16	0.14	0.05	2.80
80 mg/kg				0.07	2.00
40 mg/kg				0.18	0.78

a.FTV, fractional tumor volume = (mean final tumor volume in the experimental therapeutic group of MPH alone or TMZ alone)/(mean final tumor volume in the control group).
b.Expected FTV = (mean FTV of MPH) × (mean FTV of TMZ). Due to no significant tumor growth inhibition of MPH alone observed, the experimental data from the group of MPH at 160 mg/kg were used to calculate the combination ratio of each group.
c.Observed FTV = (final tumor volume in the combination group) / (final tumor volume in the control group).
d.Combination ratio = (Expected FTV) / (Observed FTV). > 1, synergism; < 1, no synergism or addition.

Supplementary Table S3: Pharmacokinetic parameters of MPH in plasma and xenografts in the nude mice MDA-MB-436 xenograft model

Group	Tissue	T_{max} (h)	C_{max} (ng/g)	AUC_{0-t} (h*ng/g)	$AUC_{0-\infty}$ (h*ng/g)	MRT (h)	$t_{1/2}$ (h)	$(AUC_{0-t\ Xenograft})/(AUC_{0-t\ Plasma})$
40 mg/kg	Plasma	0.5	1274	6757	7380	4.47	3.00	–
	Xenograft	3	16284	153623	158483	6.18	2.78	22.74
80 mg/kg	Plasma	2	1655	13139	13202	4.90	1.71	–
	Xenograft	6	34098	288198	309390	7.47	3.37	21.93
160 mg/kg	Plasma	0.5	3939	25802	30454	8.70	6.14	–
	Xenograft	6	44685	517050	685510	11.77	6.98	20.04

Supplementary Table S4: Pharmacokinetic parameters of MPH in rats and cynomolgus monkeys after oral administration

Animals	Dose (mg/kg)	T_{max} (h)	C_{max} (ng/mL)	AUC_{0-t} (ng·h/mL)	$AUC_{0-\infty}$ (ng·h/mL)	$MRT_{0-\infty}$ (h)	$t_{1/2}$ (h)	F (%)
Rats	10	3.0 (2.0~4.0)	116 ± 39	416 ± 123	430 ± 121	3.16 ± 0.54	1.07 ± 0.15	46.4
	20	3.5 (2.0~4.0)	368 ± 157	1633 ± 643	1666 ± 639	3.54 ± 0.50	1.15 ± 0.13	91.0
	40	2.5 (2.0~4.0)	725 ± 296	4011 ± 1620	4036 ± 1620	4.28 ± 0.39	1.3 ± 0.24	111.8
Monkeys	5	2.0 (2.0~3.0)	114 ± 13	559 ± 120	576 ± 125	4.59 ± 0.54	2.16 ± 0.35	42.4
	10	3.0 (2.0~4.0)	323 ± 50	2273 ± 510	2311 ± 496	5.98 ± 0.45	2.58 ± 0.23	85.1
	20	4.0 (4.0~6.0)	608 ± 68	5677 ± 884	5712 ± 896	7.05 ± 0.71	2.7 ± 0.23	105.1

NOTE: n = 6; 3 male and 3 female animals in each group.

Supplementary Table S5: Pharmacokinetic parameters of MPH in rats and cynomolgus monkeys after i.v. bolus administration

Animals	Dose (mg/kg)	C_{max} (ng/mL)	AUC_{0-t} (ng·h/mL)	$AUC_{0-\infty}$ (ng·h/mL)	$MRT_{0-\infty}$ (h)	$t_{1/2}$ (h)	CL (L/h/ kg)	Vss (L/ kg)
Rats	5	N.A.	449 ± 49	487 ± 36	1.55± 0.04	1.25 ± 0.07	9.17 ± 0.65	14.2 ± 1.1
Monkeys	2.5	N.A.	665 ± 81	679 ± 84	2.93± 0.55	2.32 ± 0.39	3.31 ± 0.37	9.56 ± 0.76

NOTE: n = 6; 3 male and 3 female animals in each group.

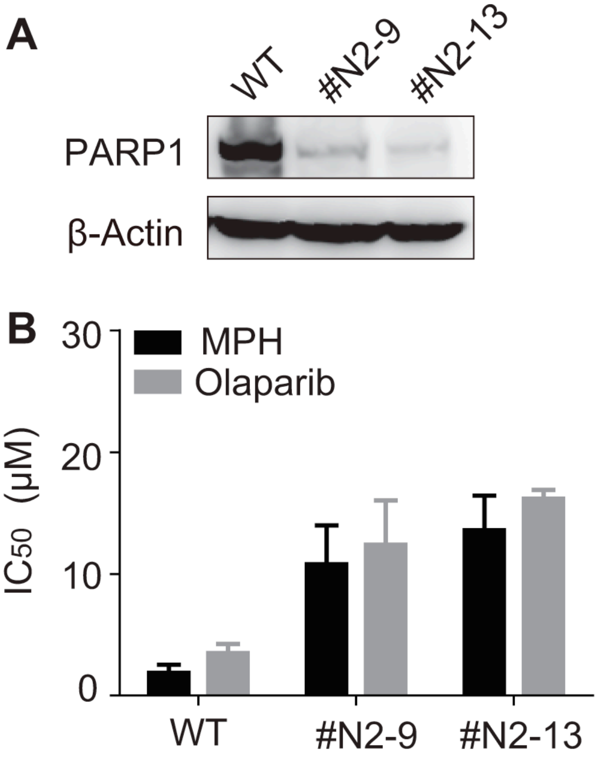

Supplementary Figure S1: Depletion of PARP1 leads to resistance to MPH. (**A**) Knockdown of PARP1. Ewing sarcoma RD-ES cells were transduced with a TALEN plasmid specifically targeting *PARP1* gene. The expression of PARP1 protein was evaluated by Western blotting and compared with wild-type (WT). #N2-9 and #N2-13 are stable PARP1-knockdown clones derived from RD-ES cells. (**B**) Loss of PARP1 protein causes MPH resistance. Cell viability was measured after treatments with MPH or AZD2281 for 72 h. Mean ± SD of IC_{50} was shown from three independent experiments.
